# Navigation-guided nasal endoscopic surgery for acute vision loss caused by fibrous dysplasia: a case report and review of literatures

**DOI:** 10.1186/s12893-021-01459-x

**Published:** 2022-01-08

**Authors:** Weijie Liu, Wenhao Jiang, Mingna Xu, Xiaozhou Hu, Mengting Wang, Haochen Jin, Yunhai Tu, Wencan Wu

**Affiliations:** Eye Hospital, Wenzhou Medical University, Wenzhou, Zhejiang Province People’s Republic of China

**Keywords:** Visual loss, Optic neuropathy, Navigation, Fibrous dysplasia

## Abstract

**Background:**

Bone fibrous dysplasia is a benign disease of bone tissue dysplasia. Vision impairment is the commonest neurological complication of craniofacial fibrous dysplasia. Most of the vision loss caused by craniofacial fibrous dysplasia is usually a gradual process. Very few present with acute visual impairment as described in our case.

**Case presentation:**

We report a patient with fibrous dysplasia presenting rapidly progressive visual loss in the left eye secondary to bone cyst formation. Transnasal endoscopic surgery guided by navigation with drainage and curettage of this bone cyst and orbital decompression resulted in progressive improvement in visual acuity that returned to normal 1 month post-operatively.

**Conclusions:**

In cases with acute visual loss due to fibrous dysplasia, emergency surgical treatment should be considered to preserve vision. In the surgical approach, navigation-guided nasal endoscopic surgery may be preferred because of its advantages.

**Supplementary Information:**

The online version contains supplementary material available at 10.1186/s12893-021-01459-x.

## Background

Bone fibrous dysplasia (FD) is a benign disease of bone tissue dysplasia, which occurs before the age of 30 years old, and the incidence rate of bone fibrous dysplasia in females is higher than that in males [1]. Combined with clinical manifestations, imaging findings and pathological examination, the diagnosis of FD is not difficult. Vision impairment is the commonest neurological complication of craniofacial fibrous dysplasia [2]. Most of the vision loss caused by craniofacial fibrous dysplasia is usually a gradual process, which develops slowly for several months to several years. By contrast, cases of acute vision loss are rare. In this case, we report a patient with fibrous dysplasia presenting rapidly progressive visual loss in the left eye secondary to bone cyst formation, we firstly use navigation system to assist nasal endoscopic decompression surgery, and achieved a good result.

Through this rare case we’d like to demonstrate that early surgical intervention will lead to a better prognosis in such patients with symptoms of optic nerve compression, while the navigation-guided transnasal endoscopic pathway we use will achieve more accurate location and less tissue damage.

## Case presentation

A patient was presented at the Outpatient department with complaints of sudden visual loss on the left eye for the duration of 4 days. No other complaints were reported, especially no headaches, orbital pain or diplopia. As for medical histories, the patient complained of having been diagnosed with FD 5 years ago with no history of trauma or other neurological symptoms. The patient’s general health was otherwise excellent.

**Fig. 1:**
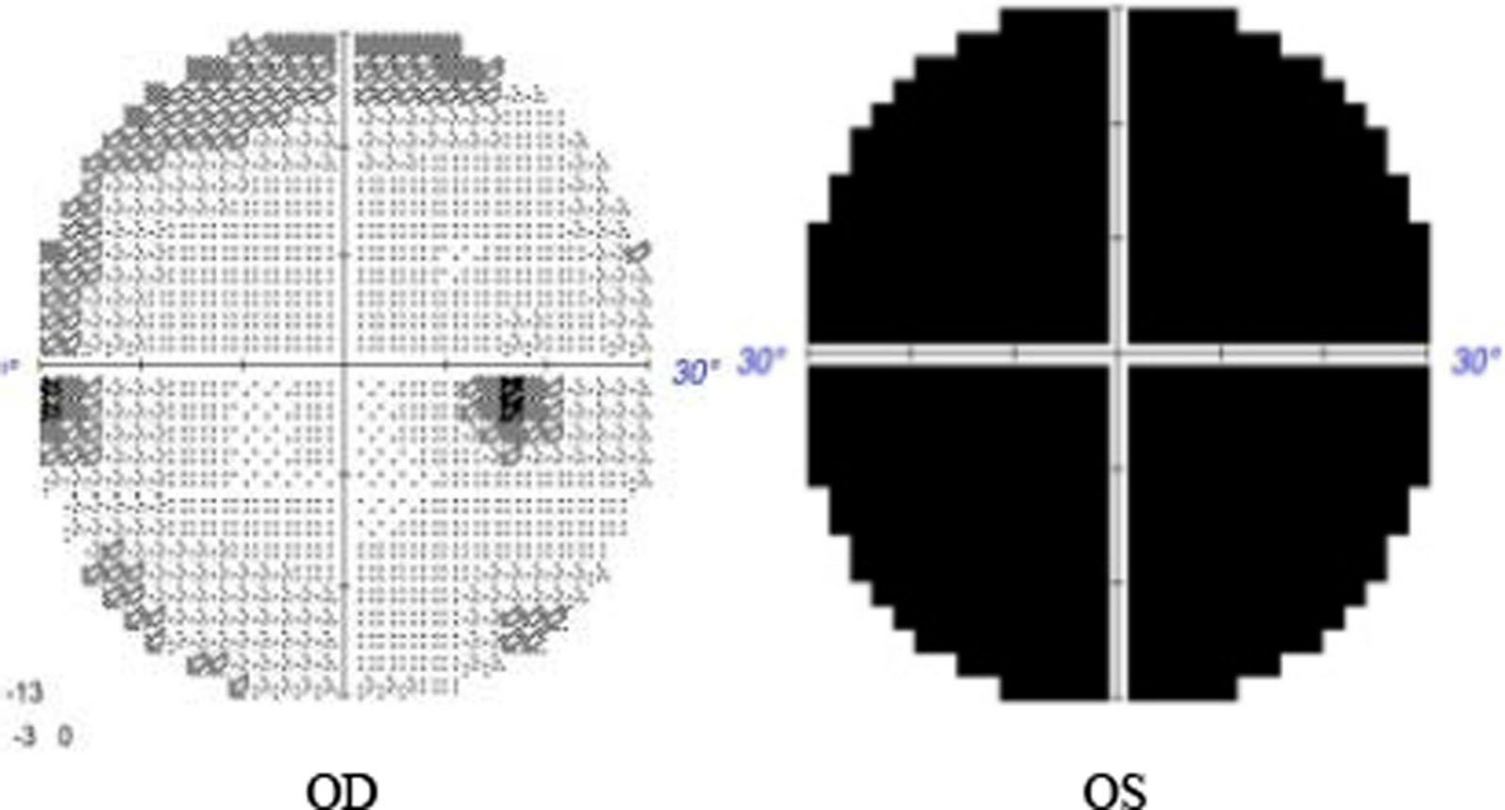
examination of visual field before surgery

**Fig. 2:**
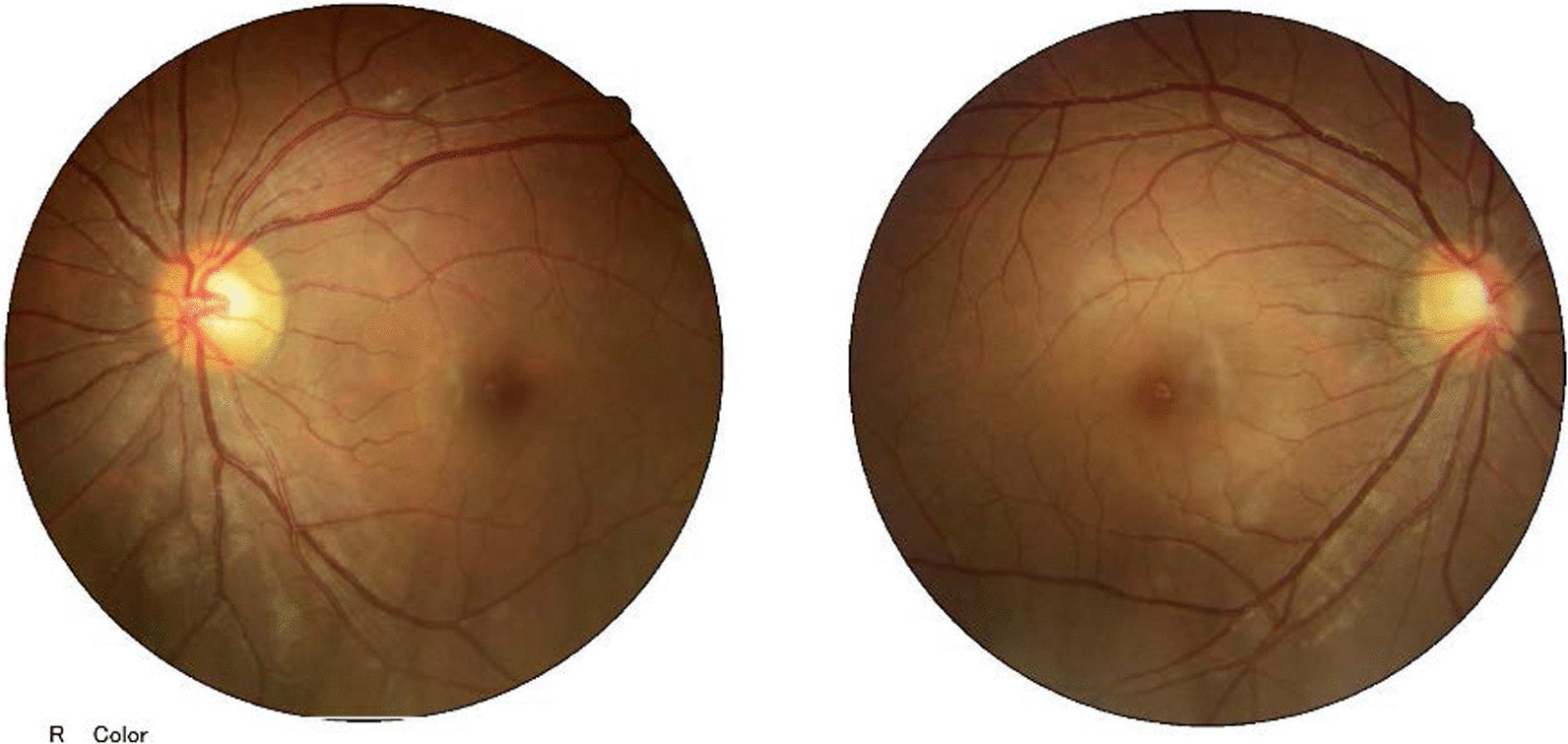
Fundus Pictures before surgery

**Fig. 3:**
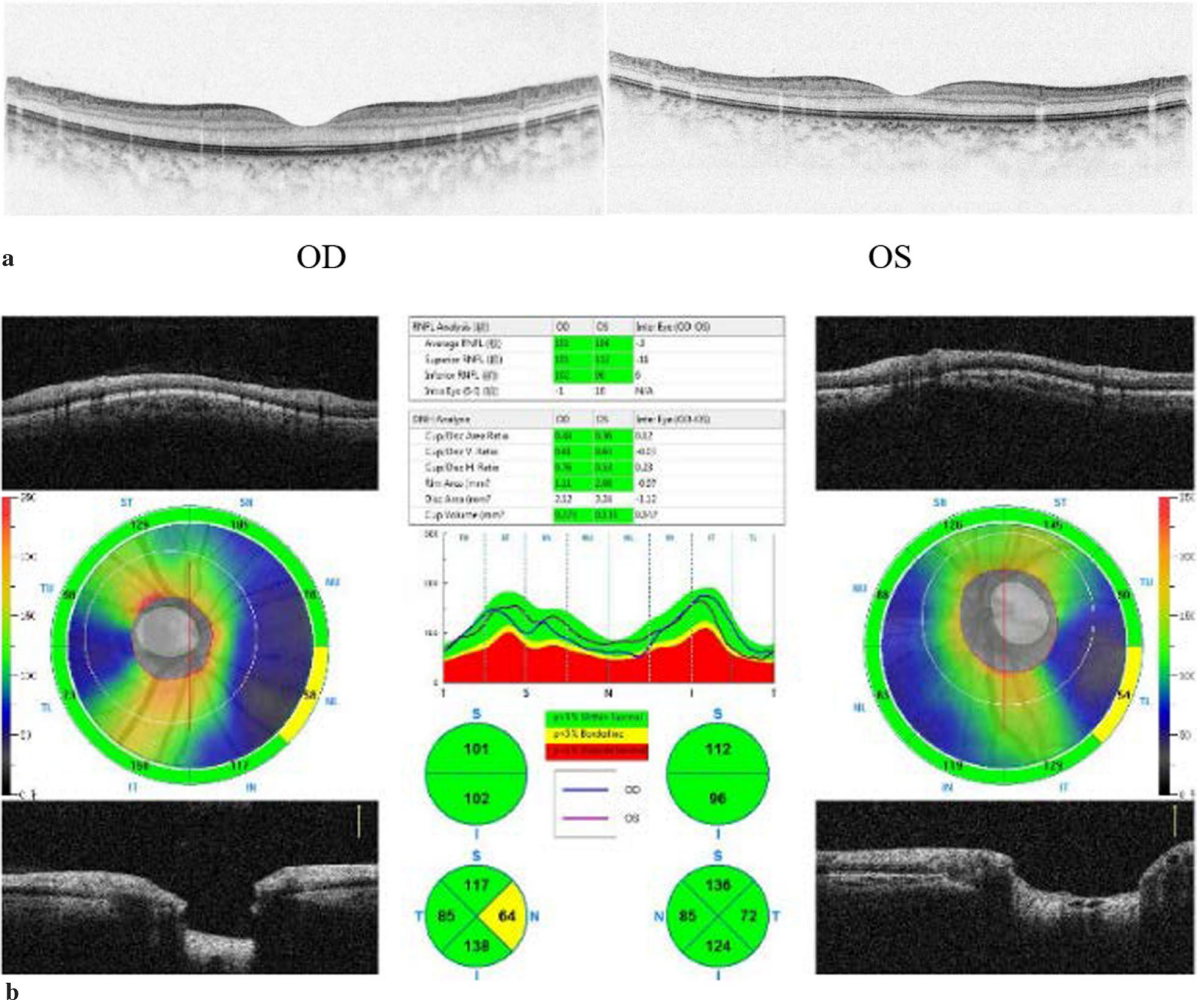
**a** Optical Coherence tomography (Macular) before surgery. **b** Optical Coherence tomography (optic disk) before surgery

**Fig. 4:**
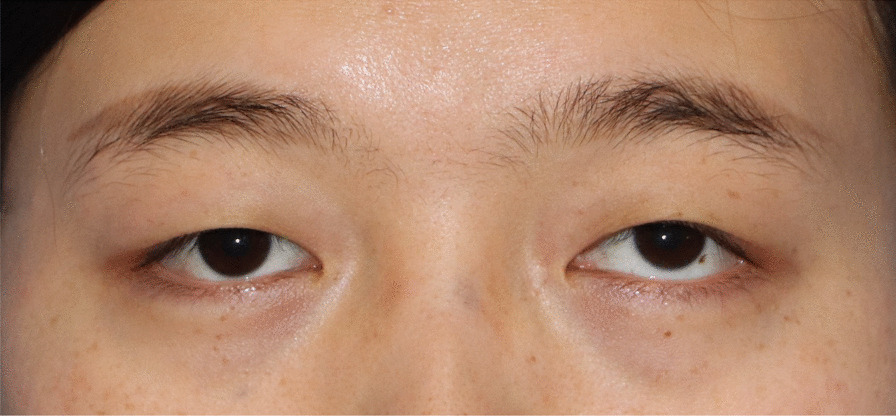
Eye position photography before surgery

**Fig. 5:**
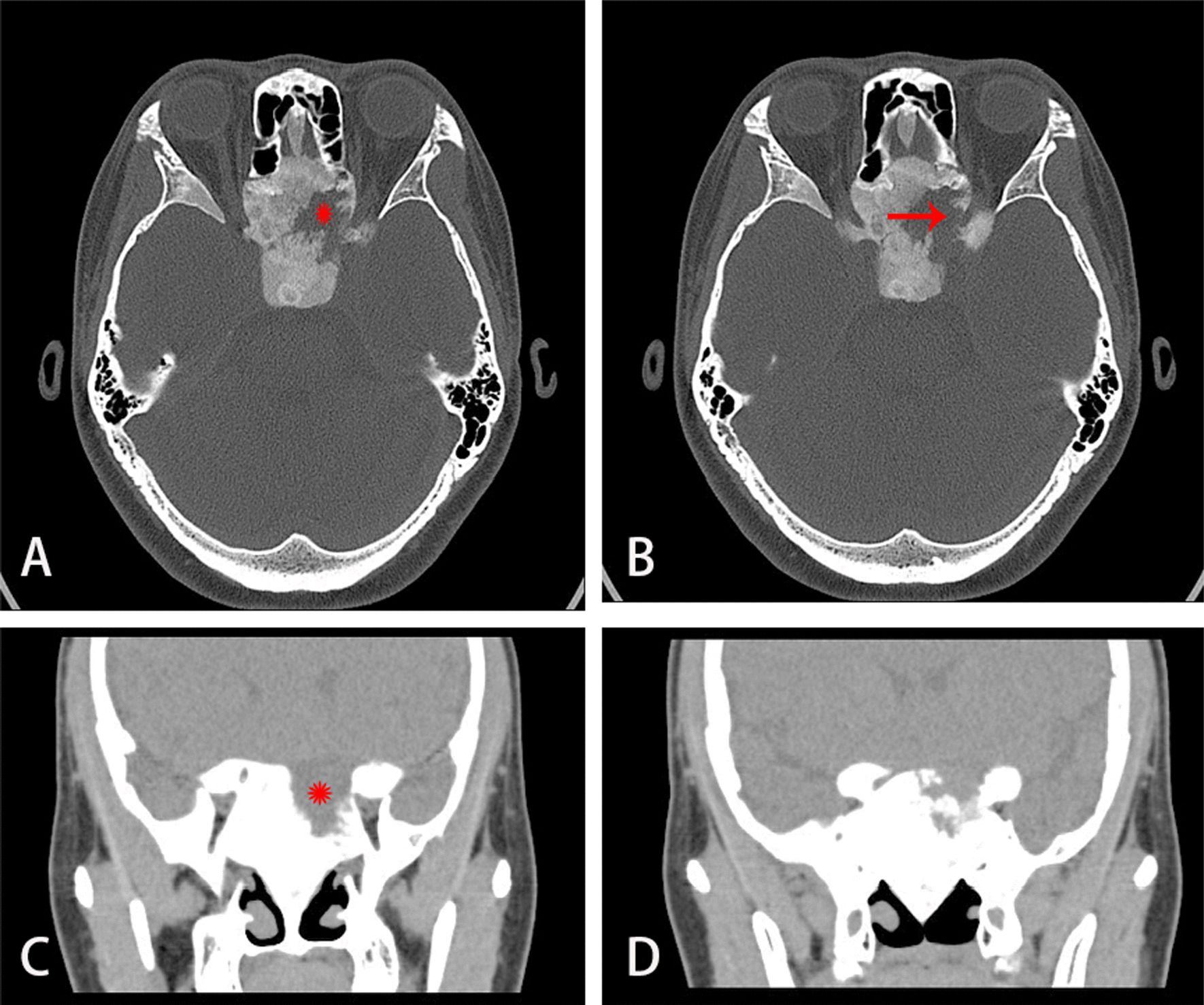
Orbital CT before surgery. **A**, **C** Orbital CT indicates fibrous dysplasia of bone with cystic degeneration (red star sign). **B** red arrow indicates optic nerve. **D** Shows the overall situation in CT

**Fig. 6:**
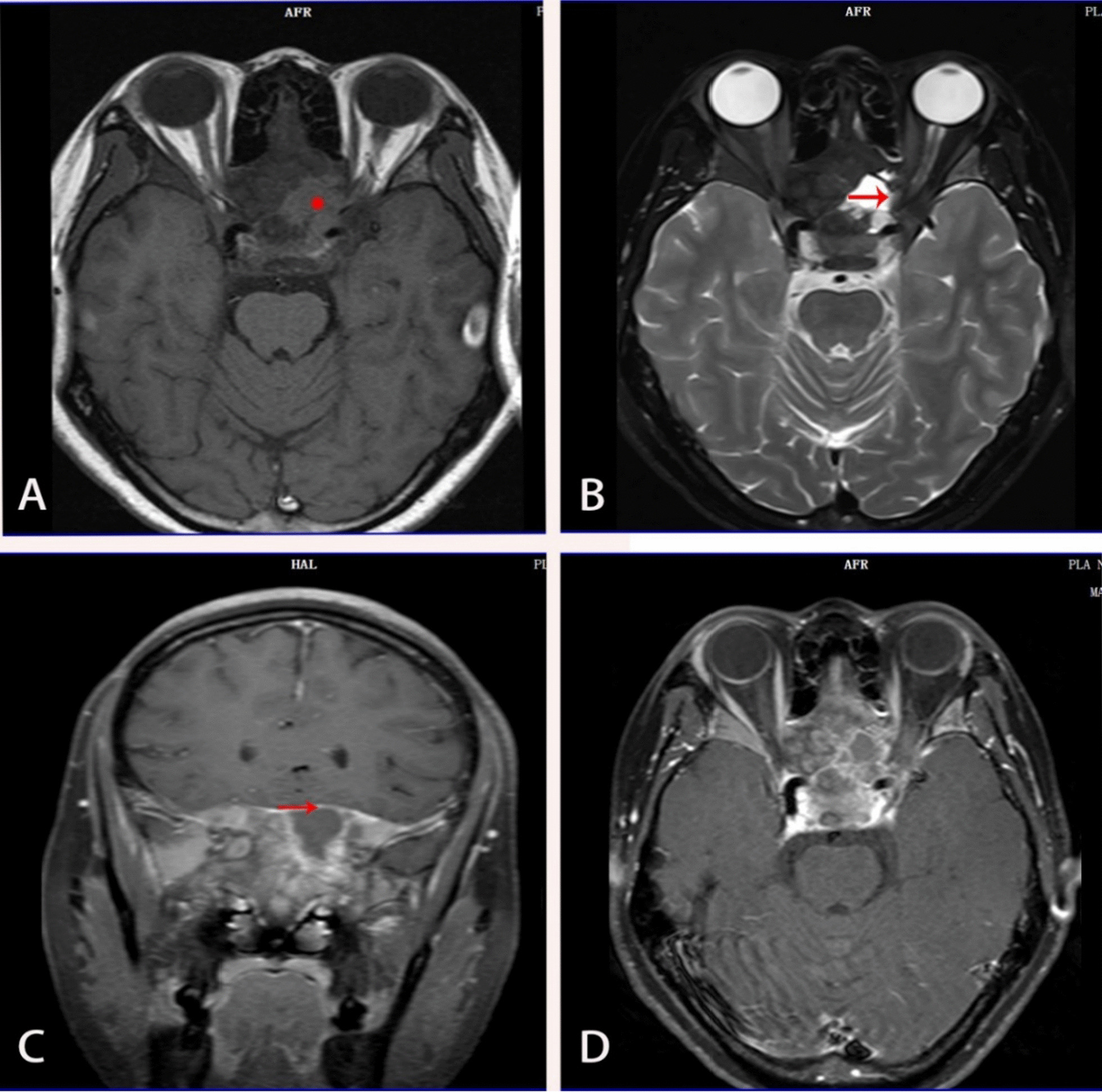
Orbital MRI before surgey. **A** Orbital MRI indicates cystic degeneration in patients with fibrous dysplasia of bone (marked by red stars). **B** The red arrow shows the optic nerve in the canal. **C** The red arrow shows the integrity of the dura mater and no collapse of the skull base. **D** Shows the overall situation in MRI

On examination, the patient’s best corrected visual acuity was 6/6 in the right eye and Hand Move/Behind Eye in the left eye, the intraocular pressure is within normal range in both eyes. The patient had no hyperemia in the conjunctiva of both eyes, the corneas were transparent and normal in size and shape. The vitreous body and the lenses were transparent and in-place. The pupil of the right eye was 3 mm in diameter, which of the left eye was approximately 5 mm in diameter. Right-eye indirect light reflex and left-eye direct light reflex were slow, with noted presence of an equivocal left relative afferent pupillary defect (RAPD). The visual field examination showed full field defect in left eye (Fixation losses: 0/13, Mean Deviation (MD): 33.83 dB P < 0.5%, Pattern Standard Deviation (PSD): 1.89 dB, Visual Field Index (VFI):0%) (Fig. 1). But the electrophysiological examinations such as visual evoked potential (VEP) and electroretinogram (ERG) were within normal range. Both eyes’ fundal examination was normal (Fig. 2), but the images of Angio-OCT showed slightly edema and thickening of optic nerve fibers in left eye (Fig. 3). There was no impairment in extraocular movement and no other abnormalities on the ophthalmic examination except for the patient’s subtle left exotropia (Fig. 4). The computed tomography (CT) scan revealed that FD affected the bones around left ethmoid and sphenoidal sinuses with a lucent area in the ethmoid bone medial to the left optic canal consistent with a bone cyst (Fig. 5). The Magnetic resonance imaging (MRI) scan showed that this cyst was compressing the left optic nerve and meanwhile there was no skull base collapse (Fig. 6).

**Fig. 7:**
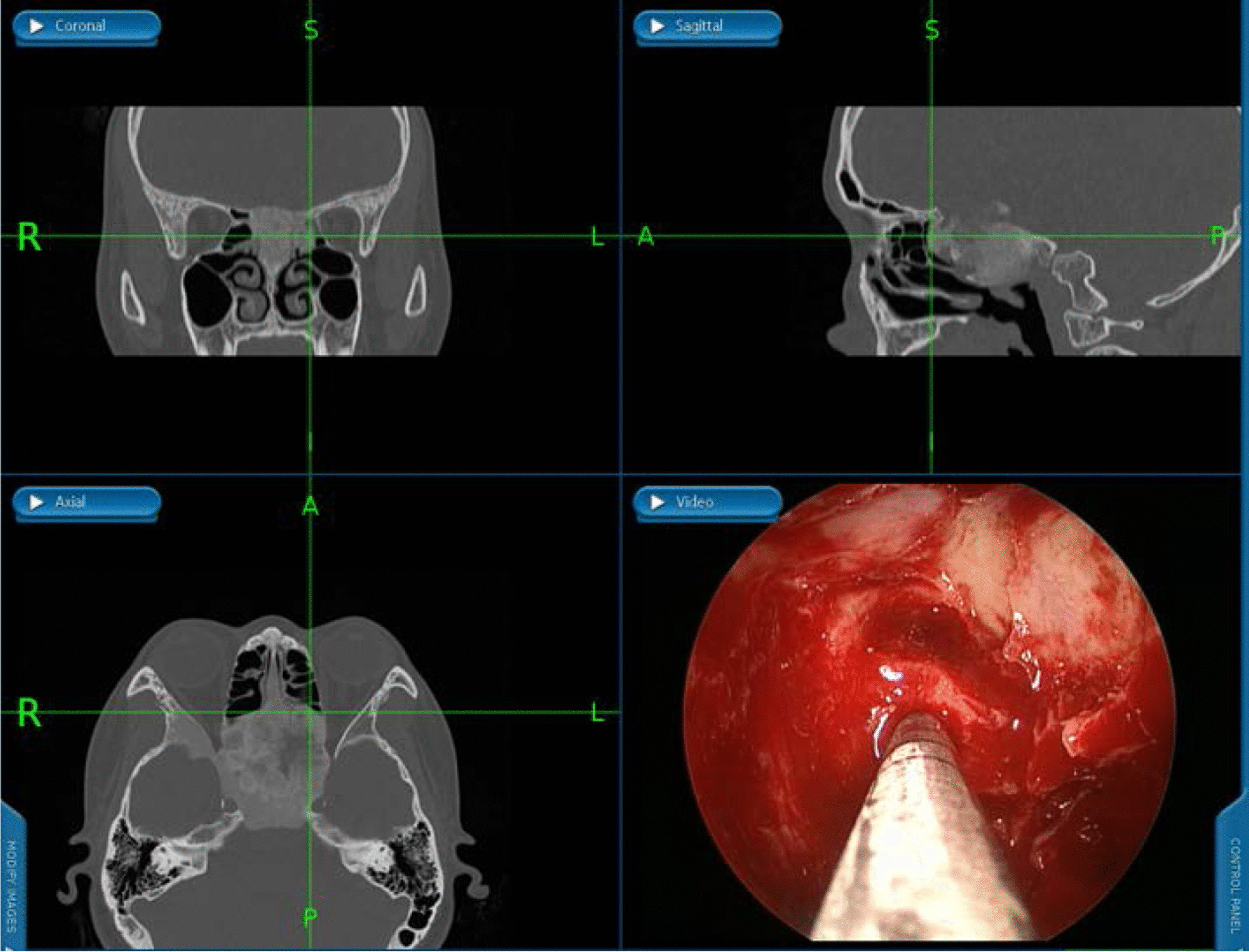
Locate the cyst under the guidance of navigation

A nasal endoscopic optic nerve decompression surgery guided by navigation of left eye was performed after preoperative evaluation with extensive hyperostosis noted in the nasal cavity and sinuses (The video of the operation can be seen in Additional file 1). After gradually removing the anterior and posterior groups of ethmoid sinus and opening of maxillary as well as frontal sinuses, we located the cyst under the guidance of navigation (Fig. 7). Then we carefully grind open the bony cystic cavity and see a large amount of cyst fluid flowing out. In order to expose the optic nerve after fully open the cystic cavity, we used navigation to locate the optic nerve and carefully removed the redundant hyperosteogeny tissue around the orbital apex and optic nerve. At the same time, the redundant hyperosteigeny tissue of skull base was removed and taken some for pathological examination. We cut open the optic sheath and periosteum of orbital apex to decompress the optic nerve, then gelatin sponges with injectable mouse nerve growth factor, triamcinolone and Dexamethasone were placed on where the decompression was performed. A histopathology examination revealed typical features of FD and there were no malignant features in any of the specimens.

**Fig. 8:**
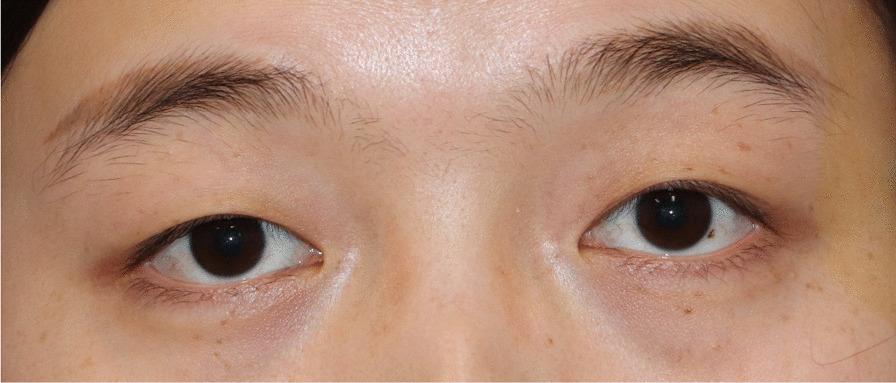
Eye position photography after surgery

**Fig. 9:**
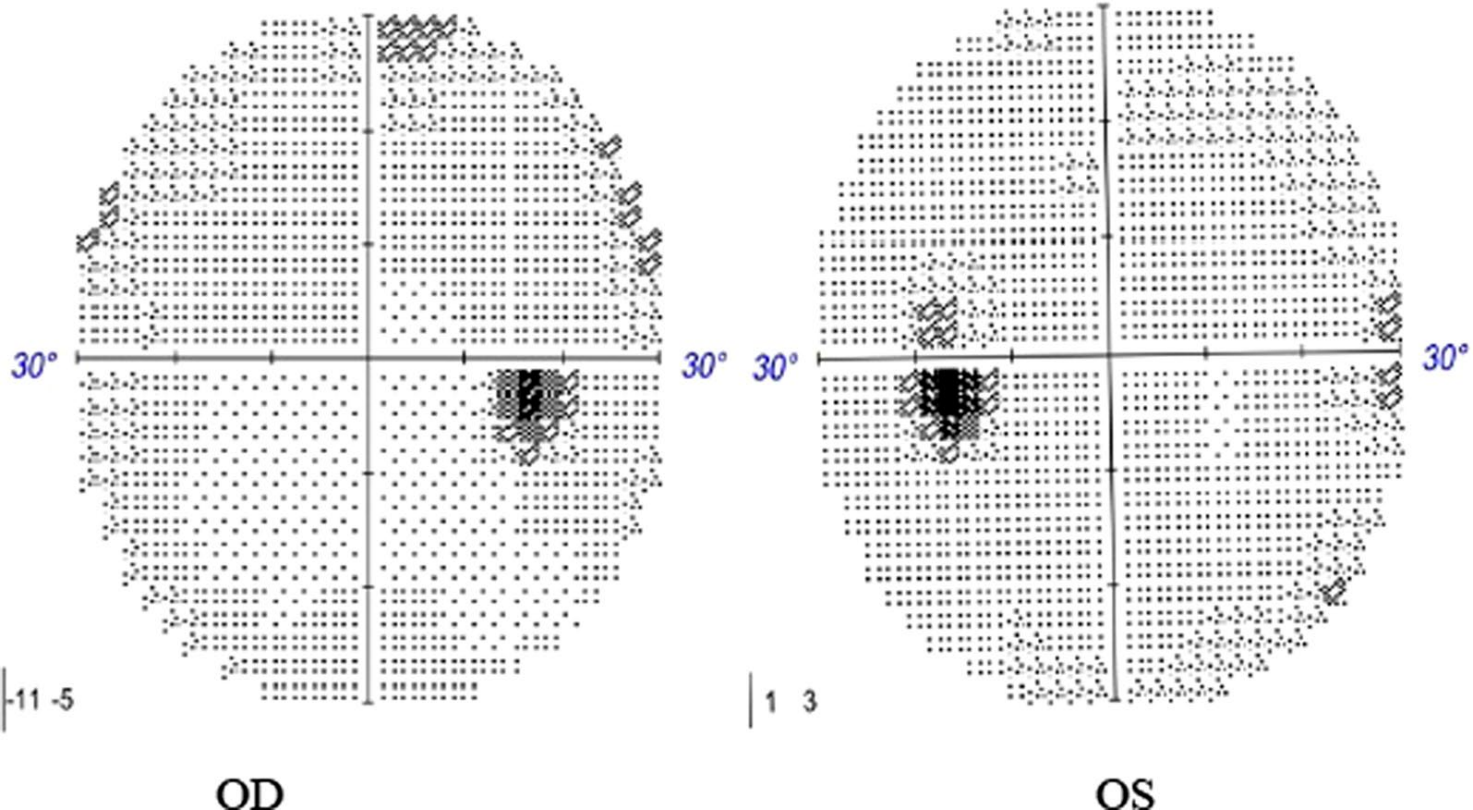
examination of visual field after surgery

**Fig. 10:**
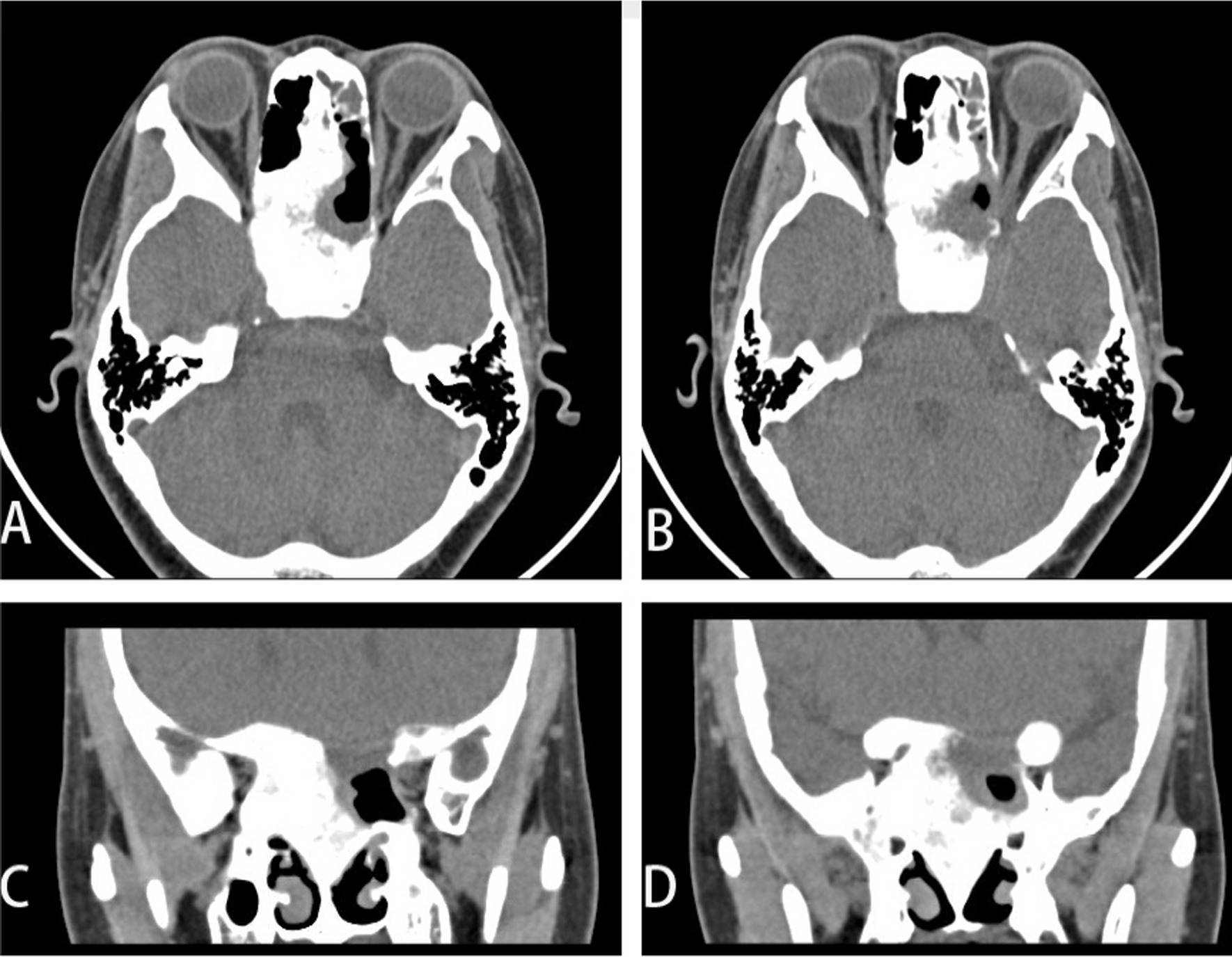
Orbital CT after surgery. **A**, **B**, **C**, **D** showed that the cystic lesions on the left side were cleared from different angles

Postoperative recovery proceeded uneventfully. Physical examination showed her left eye returned to its normal position (Fig. 8). The patient’s left eye visual acuity progressively improved to 6/7.5 on day 7, and to 6/6 at 1 month. Both eyes’ direct light reflex and indirect light reflex became sensitive. The full visual field defect in left eye completely got right one month after surgery (Fig. 9). Post-operative CT scans revealed that the left bone cyst was completely cleared (Fig. 10). The recovery of the patient’s visual acuity was beyond the expectation. We will now continue to follow the progress of the patient’s visual acuity and FD.

## Discussion and conclusion and review of literatures

The exact etiology of FD is still unknown [3]. FD is normally divided into three types: monostotic, polyostotic (accompanied by skin pigmentation such as coffee and milk stains and endocrine disorders) and McCune-Albright syndrome [1]. According to the location to the lesion, the clinical manifestations are varies. Concerning FD that affects the skull, visual loss occurs acutely or chronically [2]. Most frequently visual impairment of FD is seen due to optic canal stenosis or bone cyst formation [4, 5], but it can also occur secondary to spontaneous hemorrhage [4, 6], mucocele formation of the sinus [4, 5] and traction of the optic nerve caused by proptosis [7]. Therefore, timely use of appropriate ways to remove the optic nerve compression and pull for the rescue of vision is very important.

When the optic nerve is oppressed by concurrent cystic degeneration or hemorrhage, which result in vision loss, an operation that decompresses this nerve, can be provided. Diah et al. reported 9 cases of cyst degeneration of craniofacial FD, 4 among them with visual disturbance. Surgery restored vision in two of the four patients, but two others were confirmed to have optic nerve compression and completely lost their vision [3]. Singh et al. also reported a patient who had suffered from deterioration of vision in the left eye for 1.5 years. Postoperatively the patient improved cosmetically, but the patient’s vision recovered marginally [8]. Reviewing the cases of acute vision loss from hours to weeks, the visual acuity of the patients recovered well after optic nerve decompression [3, 9–12]. However, patients with a gradual loss of vision over years are not able to get better visual acuity after optic nerve decompression [8, 13, 14].

As for the choice of surgical methods for optic nerve decompression, transcranial and transfacial approaches are classic and universal. Although endoscopic transnasal approaches are not used much, they have existed as early as 1991 [15]. At present, the surgical methods of endoscopic transnasal approaches are becoming more and more skillful. Although, there is little literature on the use of nasal endoscope for optic nerve decompression for the treatment of other optic nerve diseases. There are a large number of literatures about using nasal endoscope to decompress optic nerve and treat traumatic optic neuropathy (TON). Meanwhile its safety has been fully proved. Compared with traditional methods, nasal endoscope takes the advantages of several aspects, such as decreasing morbidity, preservation of olfaction, rapid recovery time, more acceptable cosmetic results with no external scars and less operative stress in a patient who may have multisystem trauma and so on [15]. DeKlotz’s group [16] observed 4 FD patients who had underwent optic nerve decompression through endoscopic transnasal approaches, the best corrected visual acuity of all patients was improved after operation, and the results of the early period showed that the visual impairment before operation was completely relieved or improved after operation. The endoscopic transnasal approach does play a new role in the treatment of these diseases, but the transcranial and transfacial approaches should not be phased out, because we still need more researches to prove much more additional benefits of transnasal endoscopic surgery.

Although follow-up observation is advocated in the treatment methods for FD, referring to the above data, it can be clearly found that surgical treatment, especially for optic nerve decompression, should be actively performed in acute vision loss caused by craniofacial fibrous dysplasia. Otherwise, the loss of vision will be irreversible. And we should carefully choose the approach of operation according to the patient's condition and wish. In this case, we firstly use navigation system to assist nasal endoscopic decompression surgery, and achieved a good result. The navigation system can help surgeons design surgical paths and accurately locate lesions, together with the surgeon's knowledge and experience, is a good guide to avoid surgical complications.

## Supplementary Information

**Additional file 1:** Video of the surgery guided by navigation.

## Data Availability

Not applicable.

## Abbreviations

FD: Fibrous dysplasia
RAPD: Relative afferent pupillary defect
MD: Mean deviation
PSD: Pattern standard deviation
VFI: Visual field index
CT: Computed tomography
MRI: Magnetic resonance imaging
VEP: Visual evoked potential
ERG: Electroretinogram
